# Theoretical analysis of the thermoelectric properties of penta-PdX2 (X = Se, Te) monolayer

**DOI:** 10.3389/fchem.2022.1061703

**Published:** 2022-11-08

**Authors:** Lei Li, Zhuqin Huang, Jinqi Xu, Haihua Huang

**Affiliations:** ^1^ Key Laboratory of Extraordinary Bond Engineering and Advanced Materials Technology (EBEAM) of Chongqing, Yangtze Normal University, Chongqing, China; ^2^ School of Materials Science and Engineering, Liaocheng University, Liaocheng, China

**Keywords:** two-dimensional material, thermoelectric material, transport property, first-principles calculation, electronic structure

## Abstract

Based on the successful fabrication of PdSe_2_ monolayers, the electronic and thermoelectric properties of pentagonal PdX_2_ (X = Se, Te) monolayers were investigated *via* first-principles calculations and the Boltzmann transport theory. The results showed that the PdX_2_ monolayer exhibits an indirect bandgap at the Perdew–Burke–Ernzerhof level, as well as electronic and thermoelectric anisotropy in the transmission directions. In the PdTe_2_ monolayer, P-doping owing to weak electron–phonon coupling is the main reason for the excellent electronic properties of the material. The low phonon velocity and short phonon lifetime decreased the thermal conductivity (*κ*_l_) of penta-PdTe_2_. In particular, the thermal conductivity of PdTe_2_ along the x and y transmission directions was 0.41 and 0.83 Wm^−1^K^−1^, respectively. Owing to the anisotropy of *κ*_l_ and electronic structures along the transmission direction of PdX_2_, an anisotropic thermoelectric quality factor *ZT* appeared in PdX_2_. The excellent electronic properties and low lattice thermal conductivity (*κ*_l_) achieved a high *ZT* of the penta-PdTe_2_ monolayer, whereas the maximum *ZT* of the p- and n-type PdTe_2_ reached 6.6 and 4.4, respectively. Thus, the results indicate PdTe_2_ as a promising thermoelectric candidate.

## 1 Introduction

Currently, the energy crisis is the greatest challenge facing the world today, and requires the rapid acquisition of technological alternatives to traditional energy sources (Tan et al., 2016; Yang et al., 2018). Based on the Seebeck and Peltier effects, thermoelectric materials can convert thermal and electrical energy, thereby providing an initial solution to address this problem (He and Tritt, 2017). We use the thermoelectric quality factor *ZT* (Zhao et al., 2016; Snyder and Snyder, 2017) to measure the conversion efficiency of a thermoelectric material, expressed as:

$$ZT=\frac{S^{2}\sigma T}{\kappa }\;,$$ (1)

where *S*, *σ*, T, and *κ* are the Seebeck coefficient, electrical conductivity, absolute temperature, and thermal conductivity, respectively. The overall thermal conductivity (*κ*) is generated by the combined effect of the lattice (*κ*_l_) and electronic (*κ*_e_) thermal conductivity. Thus, a material with excellent electrical heating should achieve exceptional electrical properties [high Seebeck coefficient *S*, electronic conductivity *σ*, and power factor (PF) = *S* ^*2*^ *σ*] and thermal properties (low thermal conductivity *κ*). In addition, electrical conductivity is counter-correlated to *S* and directly proportional to *κ*_e_ (Jonson and Mahan, 1980), demonstrating the tradeoff in thermoelectric materials with a good *ZT*.

Low-dimensional materials can achieve local quantum pegging and polarization owing to the changes in their coordination environment and, thus, work well as thermoelectric materials (Dresselhaus et al., 2007). In particular, two-dimensional (2D) materials (Buscema et al., 2013; Kumar and Schwingenschlogl, 2015; Hong et al., 2016a; Gu et al., 2016; Yoshida et al., 2016; Hippalgaonkar et al., 2017; Hu et al., 2017; Huang et al., 2019; Zhu et al., 2019), especially transition metal dichalcogenides (TMDs) (Hippalgaonkar et al., 2017; Marfoua and Hong, 2019; Ding et al., 2020; Patel et al., 2020; Tao et al., 2020; Bilc et al., 2021; Wang et al., 2021), have garnered extensive attention owing to their thermoelectric properties. TMDs exhibit various crystal structures, mainly the H (*p*-6 *m*2) and T (*p*-3*m*1) phases, which are experimentally found to have good thermal conductivity and low *ZT* (Yan et al., 2014; Hong et al., 2016b; Ma et al., 2016; Zhang et al., 2017). After the discovery of pentagonal graphene (Zhang et al., 2015), the low symmetry of the pentagonal composition has elevated the study of 2D electrothermal materials, such as pentagonal Y_2_N_4_ (Y = Pd, Ni or Pt), pentagonal Y_2_C (Y = Sb, As, P) and pentasilene (Tian et al., 2016; Liu et al., 2018a; Naseri et al., 2018; Gao et al., 2019; Gao and Wang, 2020; Liu et al., 2020; Liu et al., 2021). The discovery of the good structural stability and high carrier mobility of monoclinic pentagonal PdS_2_ by Wang et al. (2015) led researchers to conduct extensive studies on the various properties and potential applications of pentaco-MY_2_ (M = Pd, Pt; Y = Te, Se ) (Oyedele et al., 2017; Sun et al., 2018; Lan et al., 2019; Zhao et al., 2020; Raval et al., 2021; Tao et al., 2021). Lan et al. (2019) demonstrated that the *κ*_l_ along the x-(y-) transport direction for PdTe_2_, PdSe_2_, and PdS_2_ are 1.42 (5.90), 2.91 (6.62), and 4.34 (12.48) Wm^−1^K^−1^, respectively. The maximum p-type *ZT* of penta-PdX_2_ (X = S, Se, Te) along the *x*-direction are 0.85, 1.18, and 2.42, respectively, demonstrating the potential of penta-PdX_2_ monolayers as thermoelectric materials. However, penta-PdTe_2_ monolayers have not yet been synthesized experimentally. In particular, although several studies have investigated the thermoelectric properties of pristine penta-PdX_2_, the conclusion are conflicting, and the principal discussions are controversial. Therefore, a systematic and detailed investigation should be conducted on the electronic and thermoelectric properties of pristine penta-PdX_2_ (X = Se, Te).

In this study, we systematically investigated the electronic and thermoelectric transport properties of monolayer pentagonal PdSe_2_ and PdTe_2_ by combining first-principles calculations and the Boltzmann transport theory. PdX_2_ exhibited the characteristics of an indirect bandgap semiconductor. The results show that the thermoelectric performance parameters of the penta-PdX_2_ (X = Se, Te) monolayers, such as thermal conductivity, relaxation time, carrier mobility, and *ZT*, show strong anisotropy in the *x* and *y* directions due to their specific structural characteristics. Compared with PdSe_2_, the heavier atomic mass and weaker chemical bonds of PdTe_2_ achieved a lower *κ*_l_ and higher *ZT*.

## 2 Computational details

All calculations were performed based on the density functional theory with the projector-augmented plane wave method used in the Vienna *ab initio* simulation package (VASP) (Blöchl, 1994; Kresse and Furthmüller, 1996). The electron exchange-correlation energy was described by GGA-PBE (Perdew et al., 1996) and the cut-off energy was set to 500 eV (Monkhorst and Pack, 1976). The structural optimization was performed until the energy change per atom was less than 10^–5^ eV, the forces on atoms were less than 10^–4^ eV Å^−1^, all the stress components were less than 0.02 GPa, and there was a maximum displacement of $5.0\times 10^{-4}\;Å$ . The *k*-point meshes were set as 12 × 11 × 1 to ensure the energy convergence. We took a 20 Å thick vacuum slab insertion to model the periodic structure of the monolayers. We used the Boltzmann transport equation based on the relaxation time approximation to calculate the thermoelectric transport coefficients (Madsen and Singh, 2006).

*κ*_l_ is mainly dominated by anharmonic phonon–phonon scattering. According to the Boltzmann–Peierls theory and the relaxation time approximation, *κ*_l_ is computed by (Yue et al., 2022):

$$\kappa_{L}^{\alpha \beta }\left(T\right)=\frac{1}{N_{q}\Omega }\underset{q,j}{\sum }C_{q,j}\left(T\right)\upsilon_{q,j}^{\alpha }\upsilon_{q,j}^{\beta }\tau_{q,j}\left(T\right)$$ (2)

where *N*_q_ is the number of wave vectors, Ω is the unit cell volume, α and β are Cartesian coordinate directions, *C*_*q,j*_ is the specific heat capacity of the *j*th phonon branch at the crystal momentum *q*, *υ*_*q,j*_ is the phonon group velocity obtained from the harmonic phonon frequency, and *τ*_*q,j*_ is the phonon lifetime. The harmonic phonon frequency was calculated by constructing a 4 × 4 × 1 supercell based on the finite displacement method, as implemented in the Phonopy package (Togo et al., 2008). The anharmonic third-order interatomic force constants (3^rd^-IFCs) were computed by constructing a 4 × 4 × 1 supercell with the cutoff value of the fifth nearest neighbor. By combining the 2^nd^-IFCs and 3^rd^-IFCs, *κ*_l_ was obtained through the ShengBTE code (Li et al., 2014).

## 3 Results and discussion

### 3.1 Structural models and electronic properties

**FIGURE 1:**
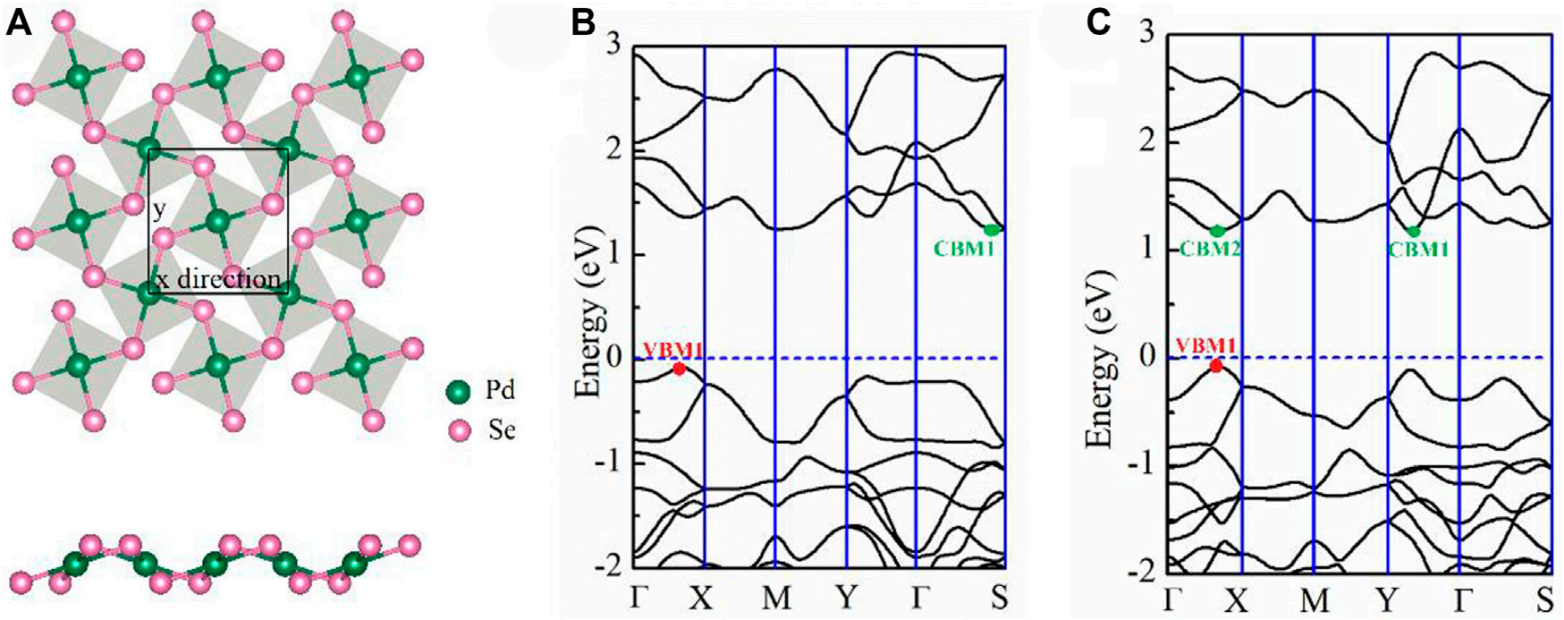
**(A)** Schematic of the top and side view of the penta-PdSe_2_ monolayer; the unit cells are marked by the black line. **(B)** Band structure of the PdSe_2_ and **(C)** PdTe_2_ monolayers. The horizontal line indicates the Fermi level. The high symmetry *k* points are: Γ(0 0 0), X (0.5 0 0), M (0.5 0.5 0), Y (0 0.5 0), and S (0.39 0.5 0).

The penta-PdX_2_ monolayer with the *P*2_1_ */c* space group (No. 14) consists entirely of pentagons. The schematic crystal structures of pentagonal PdX_2_ (X = Se, Te) are shown in Figure 1A. The diagram shows that the Pd atom is linked to four X-atoms, and that the adjacent X-atoms are connected to each other to form an anisotropic pentagonal structure. The anisotropy of pentagonal structure determines the anisotropy of the electron and transport properties. After structural optimization, the lattice parameter *a* (*b*) of penta-PdSe_2_ and penta-PdTe_2_ are 5.71 (5.90) and 6.14 (6.44) Å, respectively, which are consistent with previous theoretical calculations (Bardeen and Shockley, 1950) (see Table 1).

**TABLE 1:** Lattice parameters (a ,b), the thickness of monolayer materials (h), and bandgap (Eg) at the PBE level.

Materials	a (Å)	b (Å)	h (Å)	PdSe(Te) (Å)	Eg (eV)
PdSe_2_	5.71	5.90	1.52	2.46	1.31
PdTe_2_	6.14	6.44	1.70	2.63	1.26

To determine the electronic properties of the PdSe_2_ and PdTe_2_ monolayers, we calculated the respective energy band structures and electronic density of states (DOS), as shown in Figure 1, Supplementary Figure S1 of the Supporting Information (SI). All materials exhibited semiconducting band structures with indirect bandgaps for the penta-PdSe_2_ (1.31 eV) and PdTe_2_ (1.26 eV) monolayers. Combined with the partial DOS (Supplementary Figure S1 of SI), the conduction and valence bands were mainly composed of Pd_d and X_p orbitals. In penta-PdSe_2_, the valence band maximum (VBM) and conduction band minimum (CBM) occurred in the Γ-X and Γ-S paths, respectively, as shown in Figure 1B. In penta-PdTe_2_, the CBM values were close to the degenerate minima in the Γ-Y and Γ-X paths with a difference of 2 meV only.

### 3.2 Electronic transport properties

The carrier mobility can be calculated using the deformation potential theory, which is based on the electron–acoustic phonon scattering mechanism, and can be expressed as follows (Bardeen and Shockley, 1950):

$$\mu_{2D}=\frac{e\hbar^{3}C_{2D}}{k_{B}Tm^{*}m_{d}E_{l}^{2}}$$ (3)

where *ħ*, *k*_*B*_, *T*, and m* are the reduced Planck constant, Boltzmann constant, temperature, and effective mass along the transport direction, respectively. *m*_*d*_ is the average effective mass, decided by the effective masses along the x and y transport directions. *C*_*2D*_ represent the elastic modulus, which can be expressed as $C_{2D}=\frac{1}{s_{0}}\frac{\partial^{2}E}{\partial {\left(\frac{l}{l_{0}}\right)}^{2}}$ , where *E*, *l*, *l*_0_, and *S*_0_ are the total energy, lattice constant after and before deformation, and area of the unit cell, respectively. By fitting the band-edge curve, we can obtained the deformation potential constant *E*_*l*_.

The parameters calculated at 300 K are listed in Table 2 and include the effective mass neutrality, the elastic modulus, the deformation potential and the carrier mobility. As Table 2 shows, the anisotropy of the PdSe_2_ and PdTe_2_ structures causes them to exhibit anisotropy in all of the above parameters. In the *x*-direction, the effective mass of the holes was smaller than that of the electrons, particularly for the PdSe_2_ monolayers, which is in good agreement with their dispersive band structures (Figure 1). In the *y*-direction, the effective mass of the electrons (0.51) of PdSe_2_ was smaller than that of the holes (1.41), whereas the opposite results were found in the PdTe_2_ monolayers. The deformation potential of the holes in the monolayer materials was smaller than that of the electrons in both directions, which reflects the lower scattering rate due to the hole–acoustic phonon interaction and larger carrier mobility. As listed in Table 1, the penta-PdX_2_ monolayer exhibited high hole mobility at 300 K. For p-type doping, the small potential constant along the y transport direction indicates high hole mobility. The hole mobilities along the x(y) directions were 620 (1230) and 1063 (1817) cm^2^ V^−1^ s^−1^ for the PdSe_2_ and PdTe_2_ monolayers, respectively which mark them as showing a great advantage in electron transport.

**TABLE 2:** Calculated deformation potential (E_l_), elastic constant (C_2D_), effective mass of carrier (m*), average effective mass (m_d_), carrier mobility (μ), relaxation time (τ) of the electron (e) and hole (h) along different directions (D) at 300 K, and average Debye temperature*θ*_D_ (K).

	Carrier	D	C_2D_	E_l_	m*	m_d_	μ	τ(fs)	*θ*_D_
PdSe_2_	h	x	2.34	1.22	0.81	1.07	619.88	285.86	63
		y	3.83	0.84	1.41	1.07	1229.46	986.97	
	e	x	2.34	3.32	14.68	2.74	1.80	15.07	
		y	3.83	3.73	0.51	2.74	67.32	19.55	
PdTe_2_	h	x	1.44	1.01	0.54	0.84	1063.47	326.95	45
		y	3.18	0.74	1.30	0.84	1817.26	1345.03	
	e	x	1.44	2.09	0.87	1.12	115.61	57.27	
		y	3.18	1.80	1.44	1.12	207.96	170.49	

The relaxation time can be obtained using the following equation:

$$\tau =\frac{m*\mu }{e}$$ (4)

**FIGURE 2:**
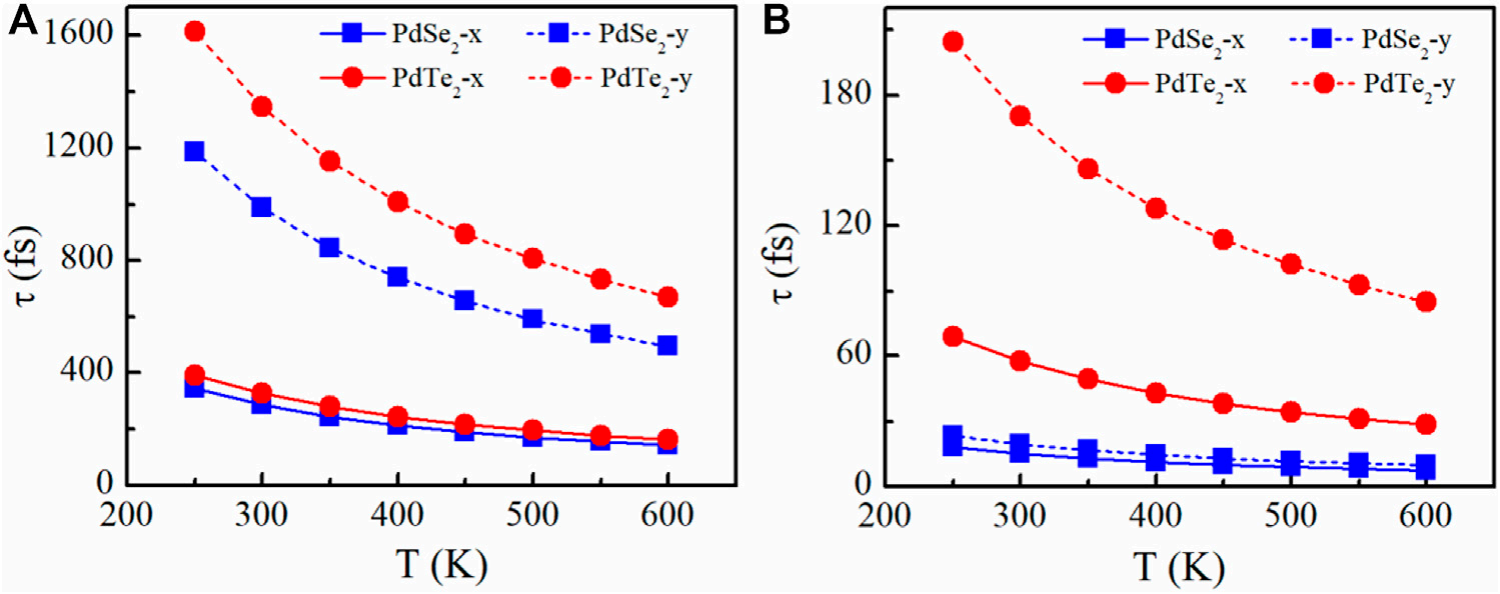
Relaxation time of the **(A)** holes and **(B)** electrons of the PdSe_2_ and PdTe_2_ monolayer along the x and y transport directions.

The temperature-dependent relaxation times of electrons and holes along the x and y transport directions is shown in Figure 2. In agreement with our predictions, the relaxation times also exhibit significant anisotropy, and the holes lifetime (Figure 2A) was significantly longer than that of the electrons because of the weaker hole phonon scattering (Figure 2B). The results demonstrated that the individual anisotropy of the relaxation times was mainly due to differences in the effective masses in x and y transport directions. Among all *p*-type candidates, The PbTe_2_ monolayer had the largest amount of relaxation time variation with temperature along the *y*-direction, which is mainly due to the smallest potential constant *E*_l_ and average effective mass m* at the VBM.

The calculated *S* and *σ/τ* along the x and y transport directions at 300 K as functions of the carrier concentration (*n*) of the PdSe_2_ and PdTe_2_ monolayer are plotted in Supplementary Figures S2, S3 of SI, respectively. With increasing *n*, *S* decreased while *σ/τ* increased. The transmission coefficients S and σ can be treated with the degenerate-doped single parabolic model, where S and σ were expressed as:

$$S_{2D}=\frac{2\pi^{3}k_{B}^{2}T}{3eh^{2}n}m^{*}$$ (5)

$$\sigma =\frac{ne^{2}\tau }{m^{*}}$$ (6)

**FIGURE 3:**
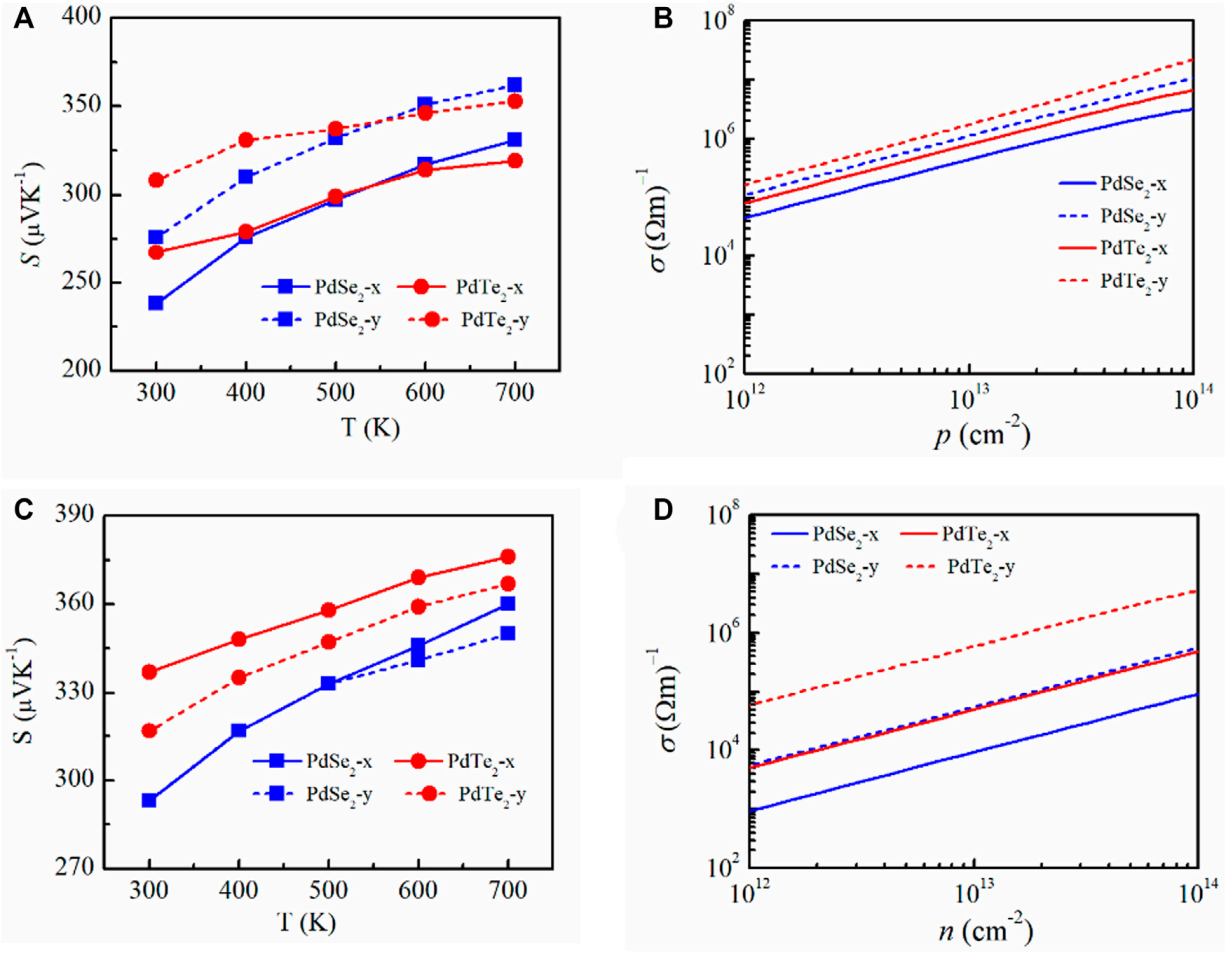
**(A,C)** Calculated Seebeck coefficient of penta-PdSe_2_ and penta-PdTe_2_ as a function of temperature and electrical conductivity. **(B,D)** Function of the carrier concentration at 300 K along the *x* and *y* directions under *p*-type (top panels) and *n*-type (bottom panels) doping.

**FIGURE 4:**
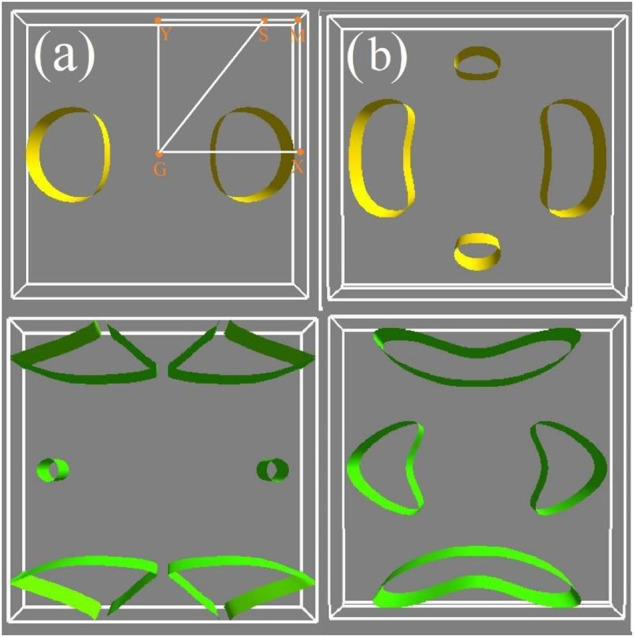
Constant energy surface of **(A)** penta-PdSe_2_ and **(B)** penta-PdTe_2_. The top images denote the highest valence with the energy of 0.1 eV less than the VBM, and the bottom images denote the lowest conduction band with the energy of 0.1 eV more than the CBM.

In the single parabolic band, *S* decreased as *n* increased, whereas σ linearly increased with *n*. Compared with p-type doping (Figure 3A), the *S* of *n*-type doping was higher for a fixed *n* (10^13^ cm^−2^). This is because the electron effective mass lies at the edge of conduction band. The isoenergy surfaces enabled analysis of the shape of the electron band. The isoenergy surfaces of the PdX_2_ monolayer were plotted (Figure 4), and the results suggested that a single pocket appear only in the Γ-X path for p-type doping, whereas multiple degenerate isoenergy pockets occurs for n-type doping. These results are consistent with previous reports on the significant enhancement of *S* owing to multiple degenerate bands at the band extrema (Xing et al., 2016; Huang et al., 2021). In *p*-type doping, the two-pocket properties (Figure 1C) increased *S* for PdTe_2_ in penta-PdX_2_ at room temperature. *S* decreased from PdSe_2_ to PdTe_2_ at temperatures above 600 K, which can be ascribed to the dual polarization effect caused by the reduced bandgap. Meanwhile, a larger hole’s effective mass increased *S* along the *y*-direction for the PdSe_2_ and PdTe_2_ monolayers. The electrical conductivity during the relaxation time is inversely proportional to *S*, (as shown in Figure 3A and C) of the SI. In addition, the higher carrier pocket degeneracy and effective mass favor a higher *S* for *n*-type doping. Among them, *n*-type doped PdTe_2_ obtained the largest *S* in the x- or *y*-direction.

Electrical conductivity was determined based on the relaxation time τ. Figures 3B,D show the electrical conductivities of the PdSe_2_ and PdTe_2_ monolayers at 300 K in *p*-type and *n*-type doping. The *p*-type electrical conductivity was higher than the *n*-type electrical conductivity because of the higher carrier mobility and longer carrier lifetime. In *p*-type doping, the electrical conductivity along the *y*-direction was larger than that along the *x*-direction, although the effective mass of the holes in the *y*-direction was higher. Similar behavior was observed for n-type doping. This trend is mainly attributed to the significantly larger elastic constant in the *y*-direction, which increased τ in all temperature ranges.

**FIGURE 5:**
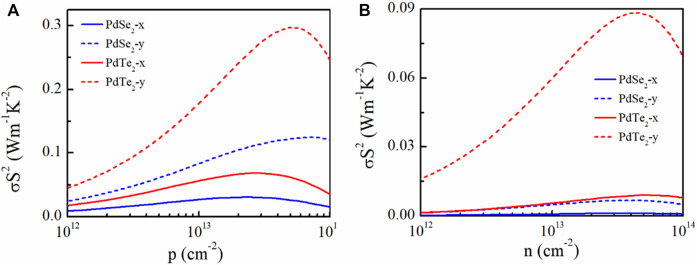
Calculated **(A)** *p*-type and **(B)** *n*-type power factor (σS^2^) of penta-PdSe_2_ and penta-PdTe_2_ as a function of the carrier concentration at 300 K along the *x*- (solid lines) and *y*- (dotted lines) directions.

PF is used to evaluate the ability of a material to convert electrical energy. The determined PF in this study is plotted in Figure 5. A higher electrical conductivity combined with a suitable *S* achieved a higher PF for p-type doping. In particular, for PdTe_2_, the highest PF during p-type and n-type doping at 300 K reached 300 mWmK^−2^ along the *y*-direction (Figure 5A) and 90 mW mK^−2^ (Figure 5B), respectively.

### 3.3 Thermal transport properties

**FIGURE 6:**
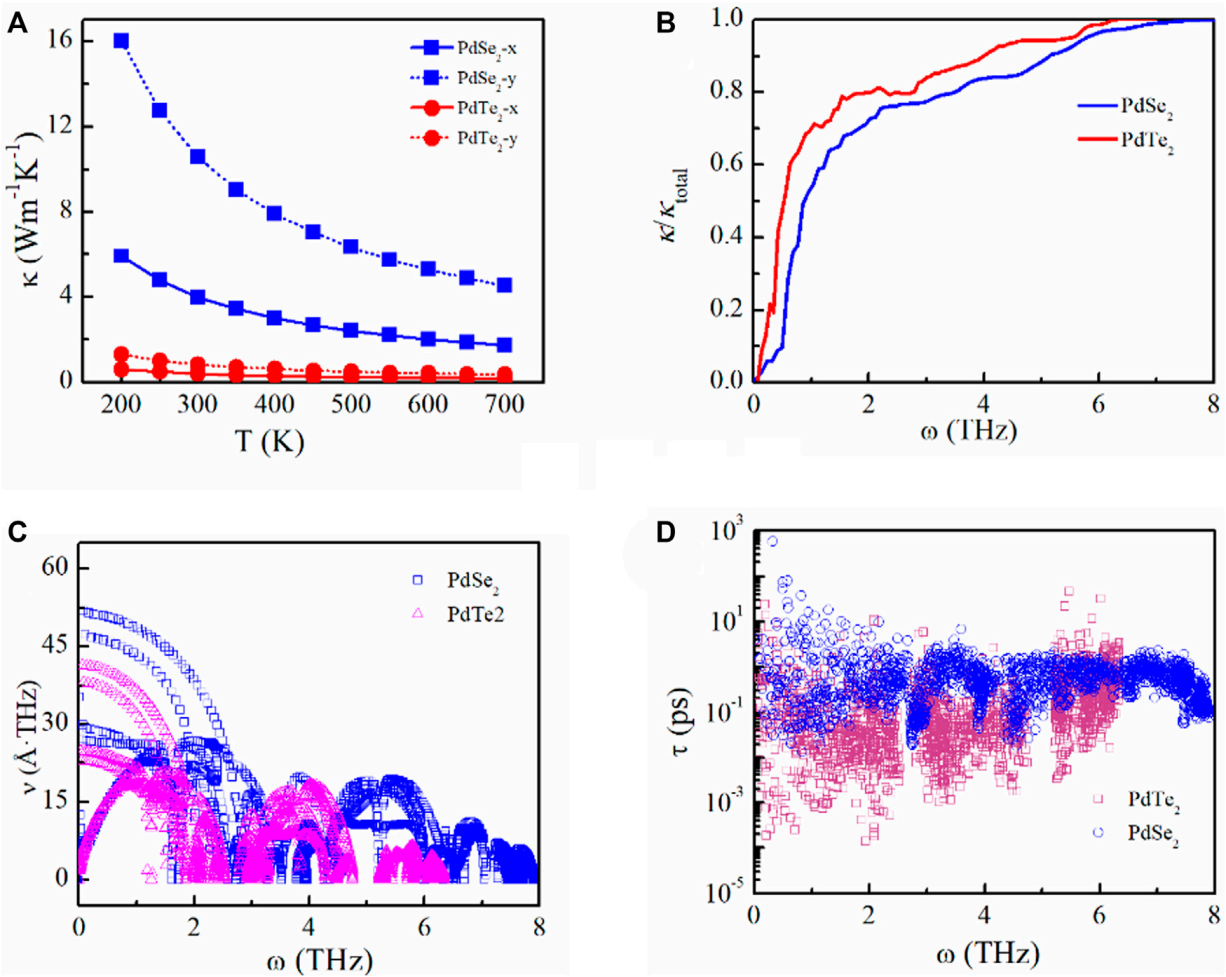
**(A)** Lattice thermal conductivity as a function of temperature, **(B)** phonon contributions toward the total lattice thermal conductivity, **(C)** phonon velocity, and **(D)** phonon lifetime of the penta-PdSe_2_ and penta-PdTe_2_ monolayers.

We analyzed the thermal transport properties of the PdX_2_ (X = Se, Te) monolayer based on the harmonic and anharmonic effects. Phonon spectra were obtained from the harmonic interatomic force constants, as shown in Supplementary Figure S4 of SI. Heavier elements and a small force constant have lower vibration frequency. The maximum phonon vibrational frequency gradually decreased from the PdSe_2_ to PdTe_2_ monolayers, indicating the suppressed phonon vibrations and shift of the optical branches toward lower energies, thereby achieving strong interactions between the phonon modes and decreasing *κ*_l_. Using the harmonic (2^nd^) and anharmonic (3^rd^) interatomic force constants, *κ*_l_ of the PdSe_2_ and PdTe_2_ monolayers were obtained, as shown in Figure 6A. In general, *κ*_l_ decreased with increasing temperature owing to the low-frequency phonons (Figure 6B) and demonstrate anisotropy along different directions. At 300 K, the calculated *κ*_*l*_ was 3.99 (10.58) and 0.41 (0.83) W m^−1^K^−1^ for PdSe_2_ and PdTe_2_, respectively, along the x(y) transport directions. These results are comparable to other thermoelectric materials with superior thermoelectric properties, such as monolayer SnSe (3 Wm^−1^K^−1^) (Zhang et al., 2016), bulk SnSe (0.62 Wm^−1^K^−1^) (Xiao et al., 2016), Ge_4_Se_3_Te (1.6 Wm^−1^K^−1^) (Huang et al., 2021), silicene (2.86 Wm^−1^K^−1^) (Peng et al., 2016), germanene (2.4 Wm^−1^K^−1^) (Peng et al., 2017), and GeSe (2.63 Wm^−1^K^−1^) (Liu et al., 2018b). Therefore, *κ*_e_ is a vital factor in the *ZT* evaluation owing to the low *κ*_l_.

To analyze *κ*_l_, we obtained the phonon group velocity and lifetime. The lower thermal conductivity is mainly attributed to the decreasing phonon velocity from PdSe_2_ to PdTe_2_, as shown in Figure 6C. Compared to the PdSe_2_ monolayer, the PdTe_2_ monolayer exhibited a shorter phonon lifetime, as shown in Figure 6D, indicating that the PdTe_2_ monolayer exhibits a considerably higher scattering rate and anharmonic feature, which can also be obtained from the phonon spectra. The scattering probability of the emission and absorption processes can be described by the frequency-dependent scattering phase space. The scattering phase spaces of the emission and absorption processes at 300 K are shown in Supplementary Figure S5 in the SI. The suppressed phonons increased the scattering phase space and enhanced the anharmonic feature, resulting in decreased thermal conductivity from PdSe_2_ to PdTe_2_ in the low-frequency region.

**FIGURE 7:**
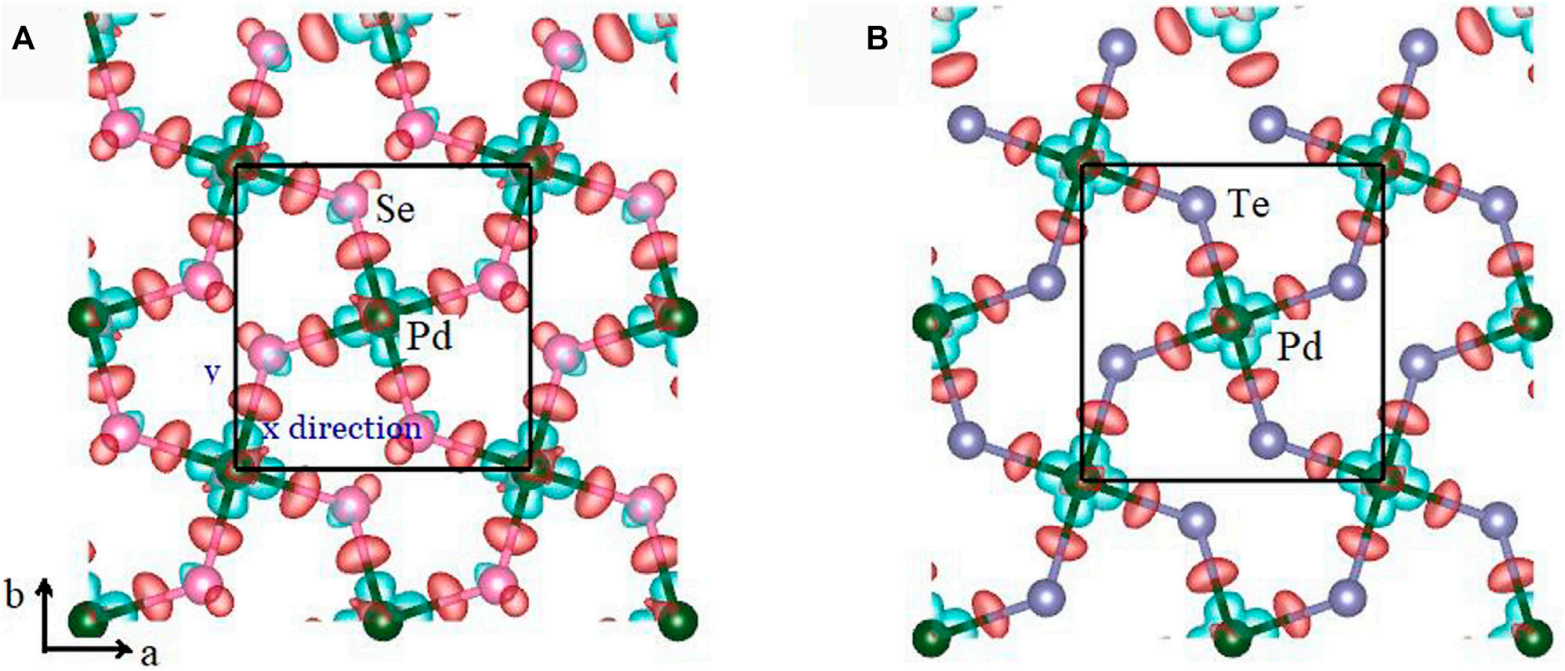
Difference of the charge density of the **(A)** penta-PdSe_2_ and **(B)** penta-PdTe_2_ monolayers, respectively. The red and green regions denote the charge accumulation and depletion, respectively. The charge density isosurfaces were set to 0.05 eÅ^−3^.

To further analyze the anharmonic feature, the difference in the charge density was computed as:

$$∆\rho =\rho_{MX2}-\rho_{M}-\rho_{X2}$$ (7)

where $\rho_{MX2}$ , $\rho_{M}$ , and $\rho_{X2}$ are the total charge density, charge density of the metal atoms, and chalcogen atoms, respectively. Lower charge density was accumulated on the Pd–Te bonds of PdTe_2_, compared to PdSe_2_, suggesting that penta-PdTe_2_ was significantly softer than PdSe_2_ with a strong anharmonic effect, as shown in Figure 7. Furthermore, the average acoustic Debye temperature (*θ*_D_) was evaluated using the formula:

$$\frac{1}{\theta_{D}^{3}}=\frac{1}{3}\left(\frac{1}{\theta_{ZA}^{3}}+\frac{1}{\theta_{TA}^{3}}+\frac{1}{\theta_{LA}^{3}}\right)$$ (8)

where *θ*_i_ (i = ZA, TA, LA) is obtained by $\theta_{i}=hv_{i,\max}/k_{B}$ , where *h*, *k*_B,_ and *v*_i,max_ are the Planck constant, Boltzmann constant, and the maximal phonon frequency of the *i*th acoustic phonon mode, respectively. The *θ*_D_ values of the PdSe_2_ and PdTe_2_ monolayers were 63 and 45 K, respectively. The low Debye temperature is due to the weak interatomic bonding of the PdTe_2_ monolayer, which suggested more phonons could participate in the scattering process, thereby reducing *κ*_l_.

### 3.4 Quality factor

The dimensionless thermoelectric *ZT* was analyzed. *κ*_*e*_ was calculated using the Wiedemann–Franz law (Jonson and Mahan, 1980):

$$\kappa_{e}=L\sigma T$$ (9)

where *L* is the Lorenza number (*L* = 2.45 × 10^–8^ WΩK^−2^). *κ*_e_ of the PdSe_2_ and PdTe_2_ monolayers at 300 K are shown in Supplementary Figure S6 of the SI, which was similar to *κ*_l_. *κ*_e_ in the *y*-direction was larger than that in the *x*-direction owing to the high electronic conductivity in the *y*-direction. The larger *κ*_e_ and *κ*_l_ in the *y*-direction also prove that the thermoelectric properties were worse along the *y*-direction than along the *x*-direction.

**FIGURE 8:**
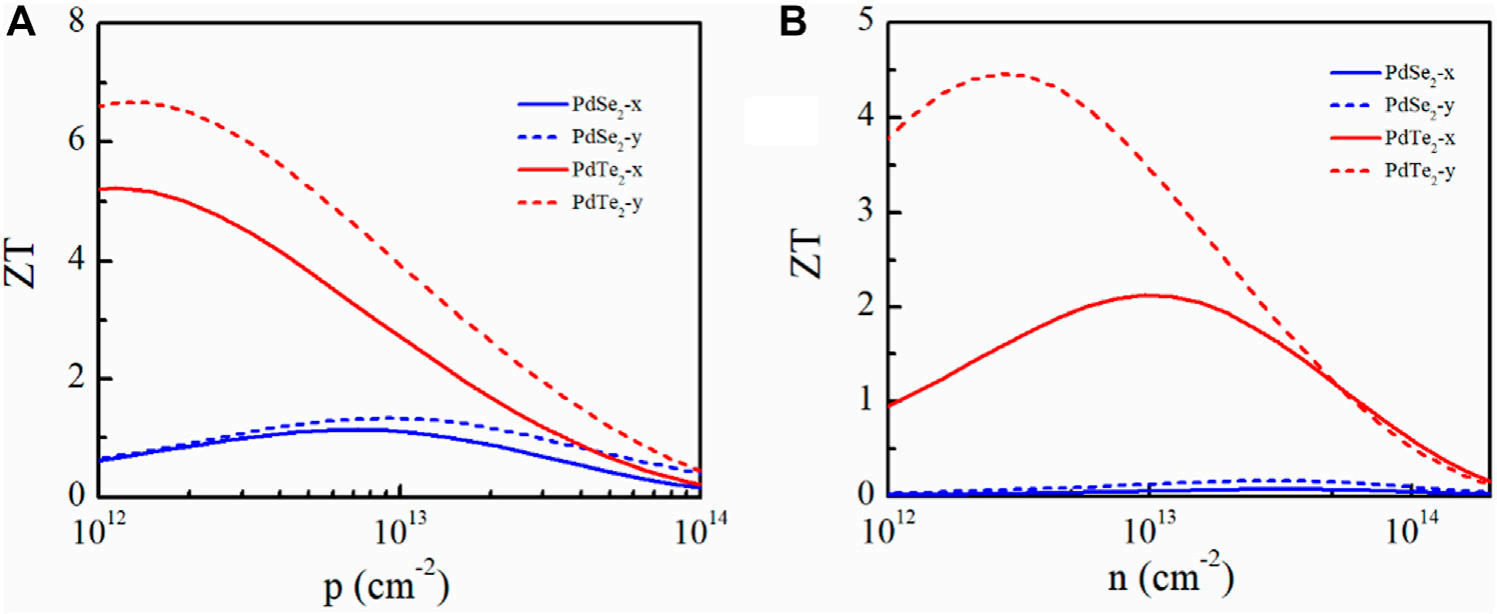
Figure of merit *ZT* of the penta-PdSe_2_ and penta-PdTe_2_ monolayers under **(A)** *p*-type and **(B)** *n*-type doping as a function of the carrier concentration at 300 K along the *x* and *y* directions.

The *ZT* values for *p*- and *n*-type doping of the monolayer materials at 300 K along the *x* and *y* directions are plotted in Figure 8. The *ZT* values of the p-type-doped PdSe_2_ and PdTe_2_ monolayers were larger than those with n-type doping because of the higher electronic conductivity. Owing to the smaller *κ*_l_ and higher PF, the PdTe_2_ monolayer obtained the largest *ZT* values, regardless of the doping type. In particular, the calculated *ZT* of the p-type- and n-type-doped PdTe_2_ monolayers along the x- (y-) direction were 5.2 (6.6) and 2.1 (4.4), demonstrating PdTe_2_ as a promising thermoelectric candidate.

## 4 Conclusion

In this study, we systematically investigated the electronic structures, electronic transport, and thermal transport properties of pentagonal PdX_2_ (X = Se, Te) monolayers using first-principles calculations and with Boltzmann transport theory. The PdSe_2_ and PdTe_2_ monolayer semiconductors had bandgaps of 1.31 and 1.26 eV, respectively. The anisotropic crystal structure of penta-PdX_2_ (X = Se, Te) achieved anisotropic transport properties. At the optimized doping concentration, the PdSe_2_ and PdTe_2_ monolayers with p-type and n-type doping along the x- (y-) directions obtained PF of up to 30 (120) and 70 (300) mW m^−1^ K^−2^, and 1 (7) and 9 (88) mW m^−1^ K^−2^, respectively. The shorter phonon lifetime of penta- PdX_2_ decreased *κ*_l_. In particular, the *κ*_l_ values were 3.99 (10.58) and 0.41 (0.83) Wm^−1^K^−1^ for PdSe_2_ and PdTe_2_ along the x- (y-) direction at 300 K, respectively. Owing to its smaller phonon group velocity and larger scattering space, PdTe_2_ exhibited lower thermal conductivity. The reasonable PF and low *κ*_l_ imply that the optimal *ZT* values of *p*-type and *n*-type-doped PdTe_2_ along the *y*-direction at the optimal doping concentrations of 6.6 and 4.4, respectively, indicating that the PdTe_2_ monolayer is a thermoelectric material with excellent properties.

## Data Availability

The original contributions presented in the study are included in the article/Supplementary Material, further inquiries can be directed to the corresponding author.
