# Supplementary figures and images for: Theoretical analysis of the thermoelectric properties of penta-PdX2 (X = Se, Te) monolayer
Source: Front Chem. 2022 Nov 8;10:1061703. doi: 10.3389/fchem.2022.1061703 (PMC9679415; doi:10.3389/fchem.2022.1061703)

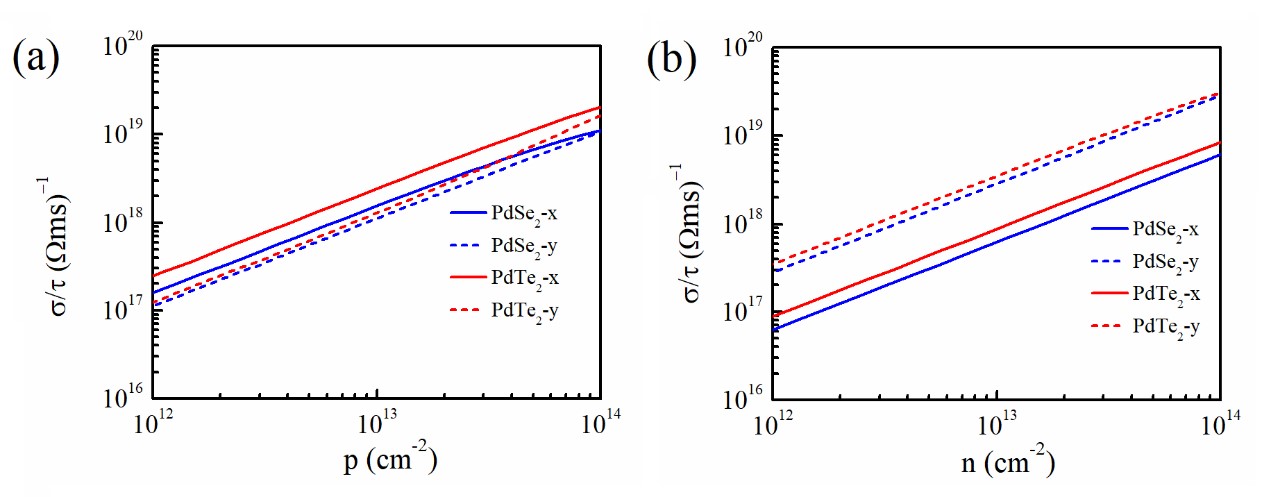

Supplement: Supplementary file 1 [file Image3.JPEG]

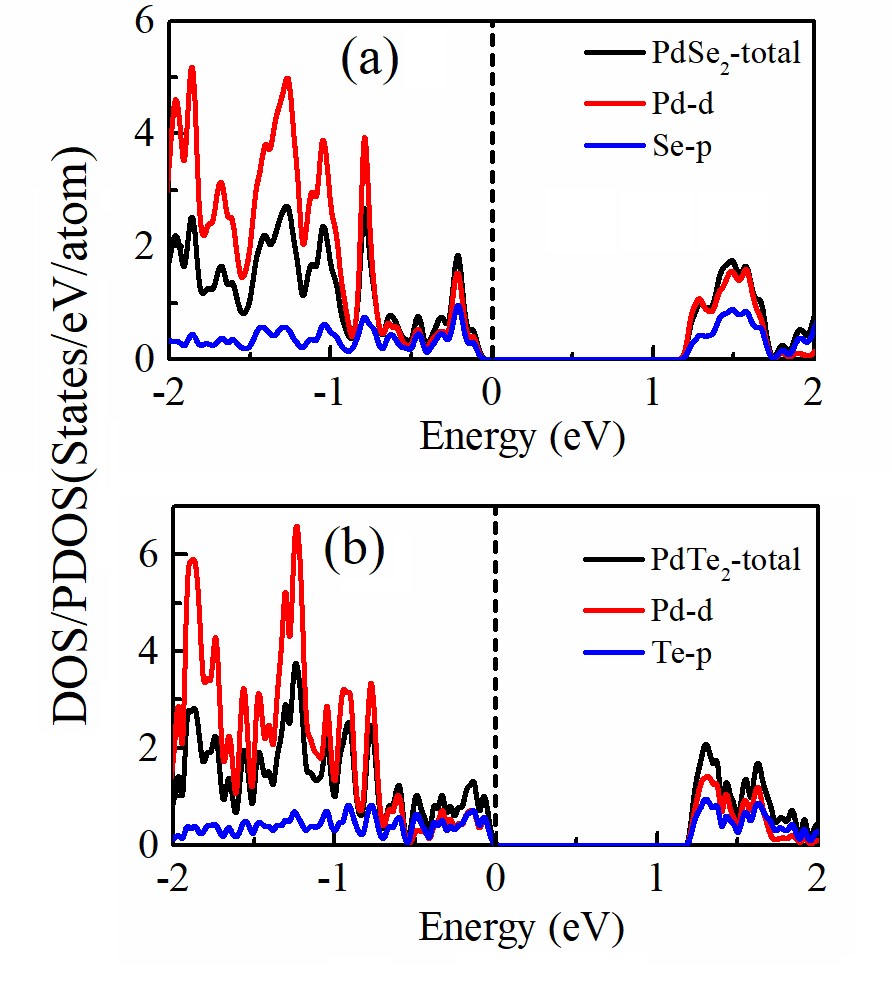

Supplement: Supplementary file 2 [file Image1.JPEG]

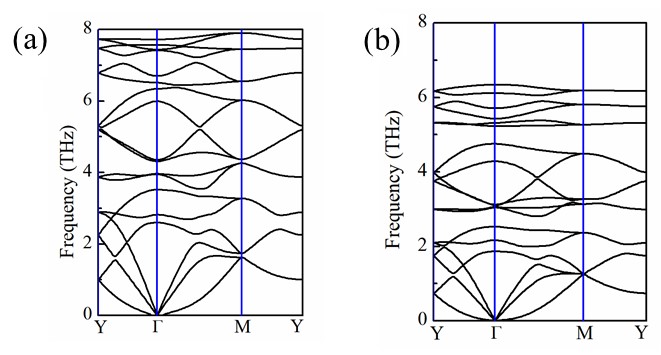

Supplement: Supplementary file 3 [file Image4.JPEG]

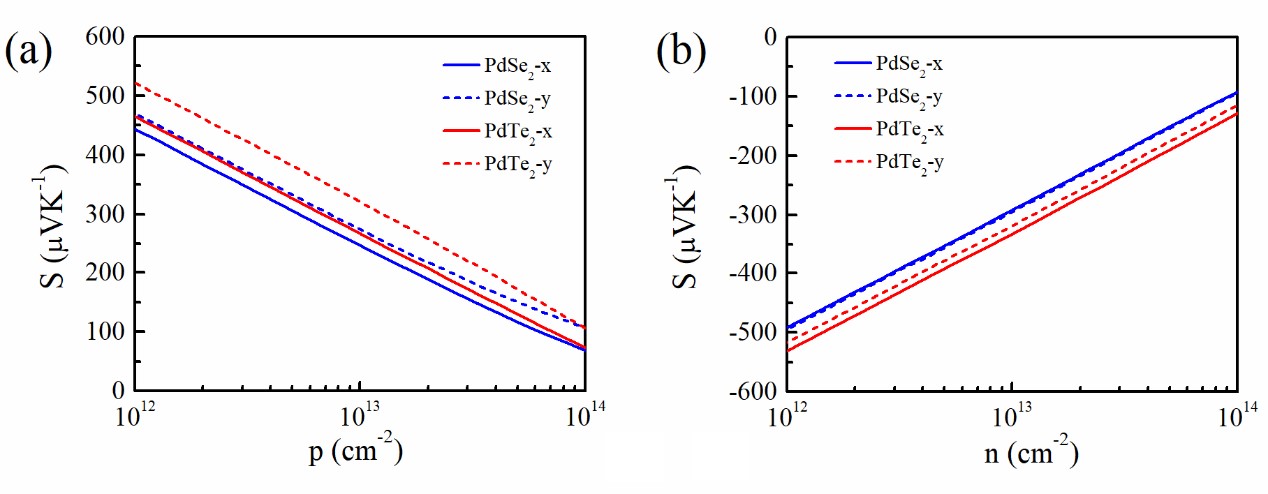

Supplement: Supplementary file 4 [file Image2.JPEG]

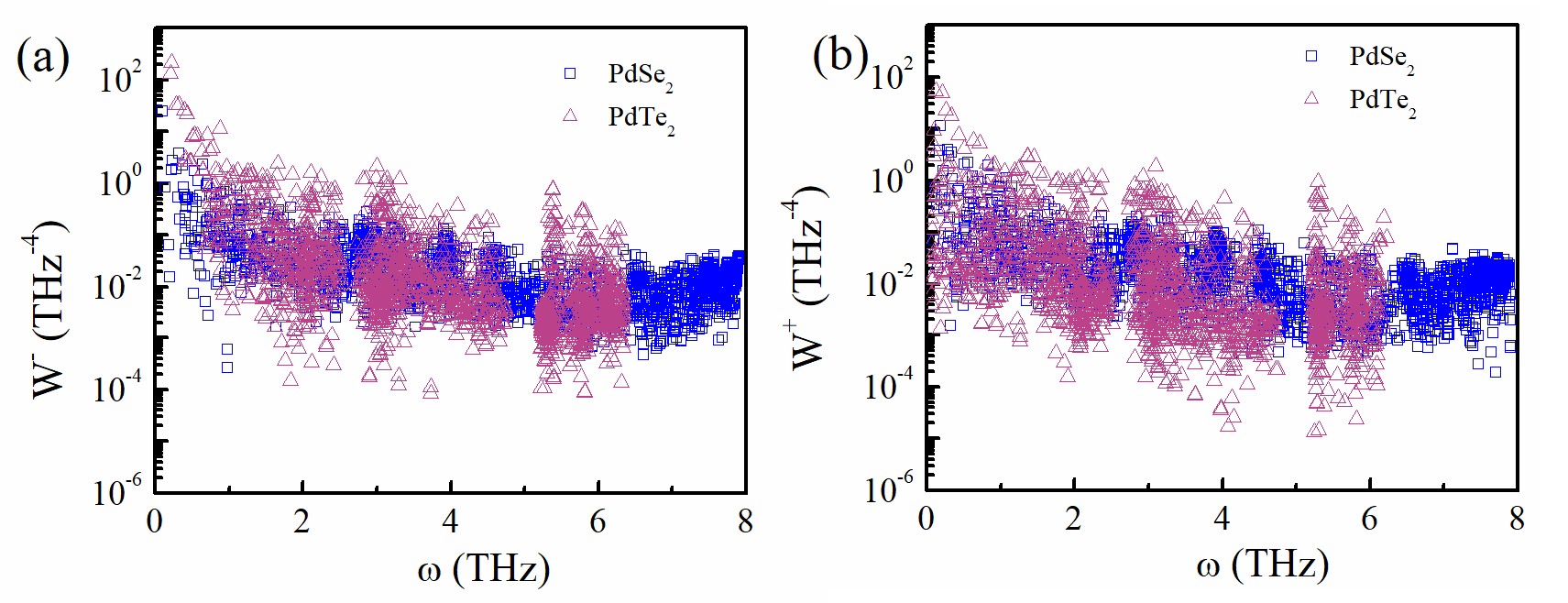

Supplement: Supplementary file 5 [file Image5.JPEG]

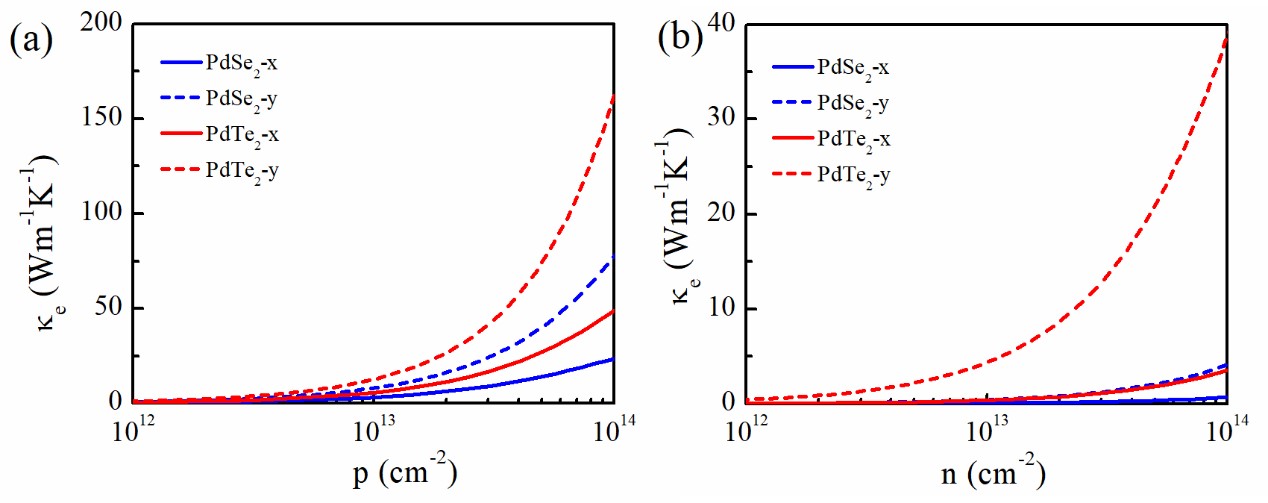

Supplement: Supplementary file 6 [file Image6.JPEG]
